# Irish medical trainees’ experiences of geographical rotations

**DOI:** 10.1007/s11845-026-04479-9

**Published:** 2026-06-11

**Authors:** Ciaran Carr, Janet O’Farrell, Rachel MacDonell, Maria Golden, John Magner, Ann O’Shaughnessy, Jess Dowling, Trevor Duffy

**Affiliations:** Royal College of Physicians of Ireland, Dublin, Ireland

**Keywords:** Geographical Rotations, Postgraduate Training, Flexible Training, Irish Medical Training

## Abstract

**Background:**

Irish Postgraduate Medical Trainees are required to relocate every three to six months in a process called geographical rotation. Rotations expose Trainees to different clinical settings and experiences. Although Trainees often request placements close to their homes, this cannot always be accommodated. The impact on Trainees is currently unknown and requires further investigation.

**Aims:**

This study aimed to investigate Irish medical Trainees’ experiences of geographical rotations.

**Methods:**

Through explanatory sequential mixed-methods, Trainees completed a questionnaire capturing their experiences of geographical rotations, including personal and professional experiences with relocating. Post-completion, participants were invited to contribute to a focus group which explored the data gathered in the initial quantitative phase. Focus group data were analysed thematically, in line with Braun and Clarke’s six-step framework.

**Results:**

The survey was completed by 261 Trainees, of whom 80%, (*n* = 225) were required to move from their primary residence while on rotation. Of these, over two thirds (67.6%, *n* = 152) continued to pay bills relating to their primary residence while taking up secondary accommodation. Through focus groups (*N* = 12), the themes of remaining connected and managing personal autonomy were prominent in the data.

**Conclusions:**

This study investigated the attitudes and experiences of Medical Trainees experiencing geographical rotations in Ireland at a national level and adds significantly to the knowledge of the Trainee experience in Ireland. This paper suggests Trainees preferences are changing, with a shift to flexible working arrangements viewed as attractive.

**Supplementary Information:**

The online version contains supplementary material available at https://doi.org/10.1007/s11845-026-04479-9.

## Background

In Ireland, becoming a doctor typically takes 10 to 15 years, depending on the training programme [1]. Training includes completion of an undergraduate degree and postgraduate training, which entails internship, Basic Specialist Training (BST) and Higher Specialist Training (HST) [1]. Postgraduate medical clinical training takes place primarily through placements in hospitals and primary care facilities [2]. Such placements occur in the form of geographical rotations, where doctors rotate through a number of separate placements to different health care facilities, depending on medical specialty, every three to six months [3]. Rotations ensure that Trainees are exposed to a variety of clinical systems and experiences while also fulfilling curriculum requirements [4].

The benefits of geographical rotations for a Trainee include exposure to different settings, gaining experience and developing professional relationships [3, 4]. Geographical rotations also benefit the clinical settings, and the broader communities Trainees are rotated to, with particular benefit to rural areas [5]. Significant differences were observed in knowledge accrued and skills learned based on geographical location, with some settings less well-resourced than others [6, 7]. Trainees in urban settings typically have access to the latest available technology and diagnostic tools as part of their training [7]. Moreover, isolated areas may be less well equipped to offer Trainees the level of supervision available in larger practices or hospitals [6].

A key challenge associated with undertaking geographical rotations is the requirement for Trainees to frequently relocate [8]. This can separate Trainees from their support networks, making it challenging to cope with the personal pressures associated with training [7]. As well as this, the current training structures are rigid, with limited opportunities to train, or work, flexibly [9]. The resultant stress has been observed to impinge on training and learning [8]. This, taken with the pre-existing stressors of training in medicine, such as long hours, making difficult decisions and coping with death and distress while maintaining compassion, all impact Trainee wellbeing [8].

The demand for flexible training, particularly with respect to less-than-full-time training (LTFTT) has increased, with improved staff retention, reduced burn-out, greater rostering flexibility, improved work–life balance and enhanced patient safety cited as benefits [9]. Among the reasons cited by Trainees for selecting LTFTT included caring responsibilities, physical and mental health, and the ability to pursue other opportunities [9]. Although a number of negative aspects have also been associated with LTFTT including Trainees feeling less well supported by supervisors, being less well able to attend conferences and academic training opportunities and reduced remuneration, consensus is that Trainees wish to train to their preferred schedule [9]. Trainees also often seek to train in their preferred location [10]. Trainees often seek to train in a setting in proximity to their home and are more likely to seek employment in a setting where they have experienced a rotation [10–12].

Limiting Trainees to a single geographical location, or geographical cohorting, has previously been observed to improve the coordination of care and quality outcomes for patients [13]. In a retrospective analysis of quality metrics collected twelve months pre- and post-implementation of geographical cohorting with positive patient outcomes observed. Significant reductions in the incidence of infections were also observed, while Trainees perceived that they were better able to manage their time [13]. Despite this, there is limited research exploring Trainee’s perceptions of geographical rotations, with no prior investigation concerned with Irish medical Trainees.

This study aimed to investigate Irish medical Trainees’ experiences of geographical rotations. While there are several reported positive and negative aspects associated with geographical rotations, the lived experience of Trainees undertaking rotations remains unknown. Furthermore, the personal impact of instances where Trainees’ location preferences are not accommodated are unknown, with the extent of the imposed financial outlay, including costs associated with accommodation and travel, as well as other factors such as extended commutes unknown.

## Methods

An explanatory sequential mixed-methods design was employed. This design involves an initial quantitative phase followed by a qualitative phase to explain and expand upon quantitative findings [14]. This approach was selected due to limited empirical understanding of medical trainees’ experiences of geographical rotation. Quantitative findings provided a broad overview of trainees’ experiences, while the qualitative phase enabled in-depth exploration of issues identified in the survey.

The study was underpinned by a pragmatic paradigm, allowing methods to be selected based on their utility for addressing the research question [14]. This approach supports the commensurability of qualitative and quantitative methods in knowledge generation and shared meaning-making [15].

All Basic Specialist Trainees (BSTs) and Higher Specialist Trainees (HSTs) at the time of data collection were invited to participate (*N* = 1,763). Participants were recruited via an email invitation distributed through RCPI communication channels. Participation was voluntary.

Survey data were received from 261 Trainees across BST and HST, representing a response rate of 15%. Data was collected at two time points. Participants were asked to complete a questionnaire on their experiences of geographical rotation, relocation, and commuting (Time 1). Quantitative analysis was descriptive and exploratory, reflecting the study’s aim to characterise trainees’ experiences. Frequencies and percentages were calculated for all variables. Analysis was conducted using IBM SPSS 20.

Participants in the qualitative phase were drawn directly from the quantitative survey cohort. At the conclusion of the questionnaire, survey participants were offered the opportunity to provide a contact email if they wished to contribute to a focus group (Time 2). This approach aligned with the explanatory aim of elaborating quantitative findings rather than generating a separate, representative qualitative sample [13]. The qualitative sample reflected a range of training levels and personal circumstances. A total of sixteen Trainees registered an interest in participating, of whom twelve agreed to participate. Two focus groups were facilitated with these twelve Trainees participating (*N* = 12). Participation was voluntary, and the sample represents a convenience, self-selected cohort.

Prior to the quantitative phase, a semi structured focus group schedule was drafted. This was further developed following preliminary analysis of survey responses, consistent with explanatory sequential mixed methods methodology [14]. Survey findings informed the inclusion and ordering of topics, with minor adjustments made during data collection to allow participants to expand upon key findings, particularly those relating to relocation, commuting, family life, and financial implications. While not formally piloted, the schedule was reviewed by the research team and refined to ensure clarity and relevance. The schedule is provided as appendix 1. Focus groups were facilitated on Microsoft Teams in February 2023. Ethical approval was granted by RCPI’s Research Ethics Committee Standard Application Form (RECSAF) 184.

Focus groups were facilitated by one of the authors, with the lead author present taking field notes. Both focus groups were recorded for later transcription and participants were anonymised during the transcription process. Qualitative data was analysed using NVIVO for data management and coding. Thematic analysis was the chosen approach to analyse the focus groups as it offered a comprehensive method of presenting the data generated [13]. Analysis was informed by interpretivist orientation of constructivism, in which the social, cultural, and structural contexts that influenced Trainees’ experiences were emphasised, thus enabling the development of knowledge that is constructed through interactions between the researcher and the research participants [13]. Data adequacy was assessed in relation to the study’s explanatory aims rather than data saturation. The focus groups generated rich, detailed accounts that enabled meaningful elaboration of key survey findings, providing sufficient depth to address the research questions [15]. In doing so, the adequacy of the qualitative data was evaluated through conceptual depth and theoretical sufficiency achieved via iterative engagement with the data, rather than reliance on saturation as a procedural endpoint [15]. To assess the extent to which the authors agreed on the themes generated, consensus was employed and achieved through discussion, reflexive engagement and shared sense-making [16]. The specific thematic analysis employed was Braun and Clarke’s six-step framework because of the clarity and usability of the framework for doing thematic analysis [13]. The six steps outlined by Braun and Clarke are detailed below;

1. Familiarising yourself with your data.
2. Generating initial codes.
3. Searching for themes.
4. Reviewing themes.
5. Defining and naming themes.
6. Producing the analysis.

### Researcher reflexivity

All authors and those who participated in this study are affiliated with the Royal College of Physicians of Ireland. This positioning may have influenced participant engagement and interpretation of findings. To mitigate potential power imbalances, participation was voluntary, confidentiality was emphasised, and data were anonymised at the point of transcription. Reflexive discussions were held throughout analysis to consider how researcher positioning and assumptions may have shaped co-construction of meaning. Decisions regarding the interpretation of the data were made through consensus to ensure the community voice was primary.

## Results

The demographic details of survey respondents collected at Time 1 and are presented in Table 1. The majority of respondents were female (*n* = 180, 69%), HSTs (*n* = 186, 71.3%) and in training (*n* = 217, 83.1%).

**Table 1:** Demographic details of respondent Trainees

Gender		*N* (%)
	Female	180 (69%)
	Male	81 (31%)
	I prefer to self-describe	-
Status		
	HST Trainee	186 (71.3%)
	BST Trainee	75 (28.7%)
Training Status		
	In training	217 (83.1%)
	On out of clinical programme experience	37 (14.2%)
	On a leave of absence	7 (2.7%)
Total		261 (100%)

Data on Trainee relocation was also collected at Time 1 and is presented in Table 2. A total of 86.2% (*n* = 225) Trainees detailed that they were required to move from their primary residence due to rotations, of which over two thirds (67.6%, *n* = 152) continued to pay bills relating to their primary residence while taking up secondary accommodation.

**Table 2:** Relocation and geographical rotation

	Yes *N* (%)	No *N* (%)
Have you had to move from your primary residence to take up a rotation placement? (*N* = 261)	225 (86.2%)	36 (13.8%)
If so, did you continue to pay (rent, mortgage, utilities etc.) for your primary residence while living in secondary accommodation while on rotation? (*N* = 225)	152 (67.6%)	73 (32.4%)

**Fig. 1:**
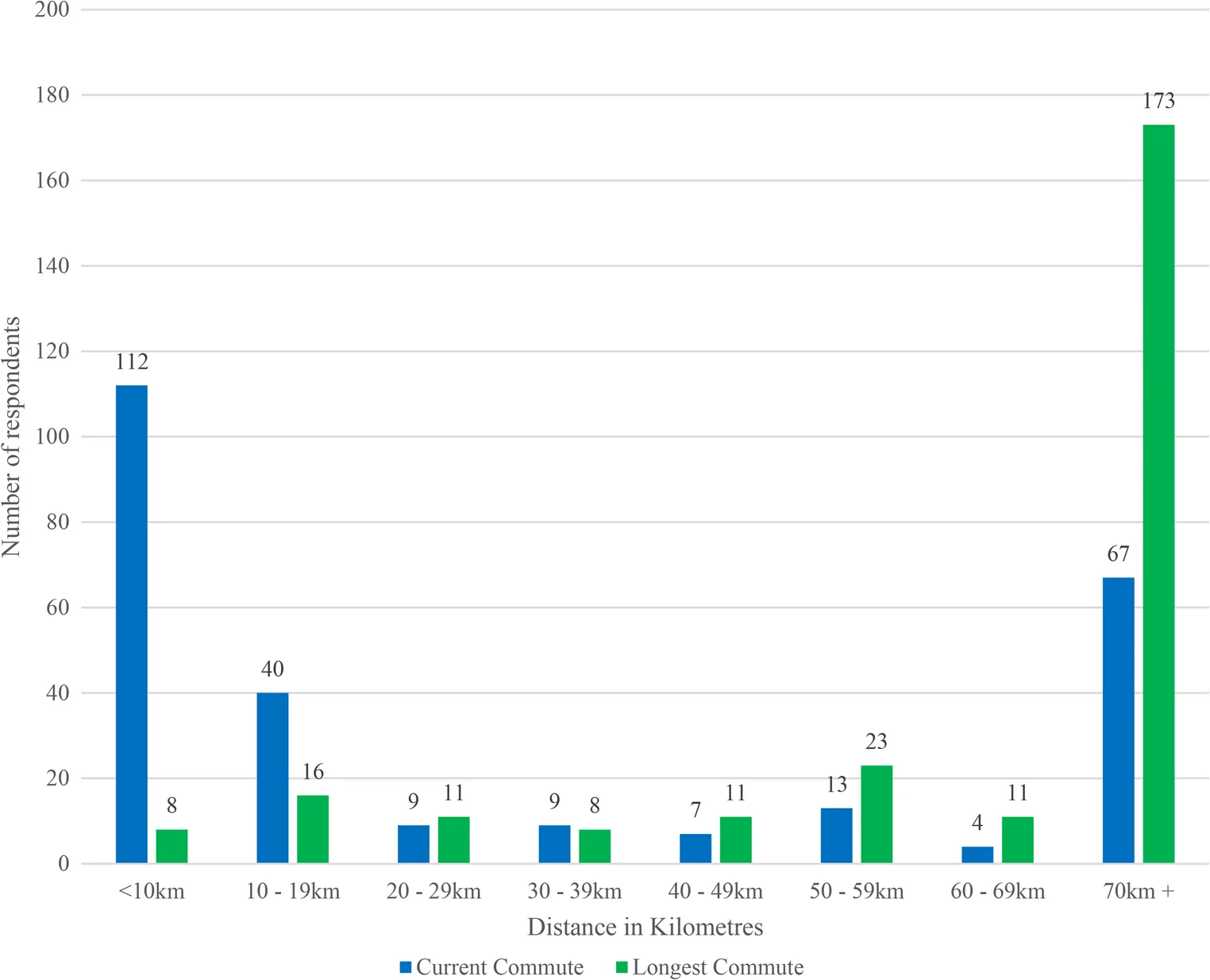
Trainee’s Current and Longest Commute in this training period

With respect to their commute, most Trainees (42.9%, *n* = 112) detailed that their current commute was less than 10 km, while 173 Trainees (66.2%) indicated that their furthest commute was greater than 70 km (see Fig. 1).

### Thematic analysis of focus groups

Focus groups were carried out at Time 2. A total of two focus groups were facilitated with twelve Trainees participating (*N* = 12). Qualitative data were presented in themes with reference to participant quotes, presented verbatim and capture the essence of the findings, providing the evidence for the themes. These were refined to the main themes and subthemes. The two main themes and five subthemes were prominent in the data and are presented in Table 3. The main themes identified were ‘Remaining Connected’ and ‘Managing Autonomy’.

**Table 3:** Presentation of themes and subthemes for Thematic Analysis

Remaining Connected	Managing Personal Autonomy
Family and the provision of care	Living arrangement
Difficulties imbedding into communities	Commuting
	The value placed on training

### Remaining connected

#### Family and the provision of care

Participants frequently discussed geographical rotations in relation to connections and the importance they placed on remaining connected. The connections discussed extended across the lifespan, with the difficulties associated with being separated from family most frequently referenced. They spoke as parents, partners and citizens, referencing the emotions associated with being separated from loved ones. Additionally, participants discussed the toll that rotating had on their personal lives.

*‘I’ve worked with people who FaceTime their children from work to say goodnight to them. It’s tragic.’* (P10, F)

The provision of childcare featured prominently, with some participants discussing being advised to employ au pair in order to support them while training due to long working hours and incompatibility with other childcare arrangements. In addition to the financial implications associated, participants discussed the intrusion, with many being required to employ live-in nannies or au pairs. They described the change to their family dynamic imposed by this, while also discussing their displeasure at being unable to be less actively involved in raising their children.

*‘I actually had kids because I wanted to raise them. I don’t want them to be in childcare.’* (P6, F)

One participant detailed their experience of being unable to personally provide care for their children. They referenced relying on others for childcare and practical support, specifically detailing their guilt at asking others to take their children to crèche. They highlighted the inflexibility of start times and the chain of events that transpire as a result.

‘*My husband isn’t a medic but travels a lot*,* so when he’s away I have to get someone to come to my house at 6:20*,* so I can leave at 6:30. And actually the guilt of asking that*,* my kids are in crèche*,* so someone has to come to mind them and then drop them to creche at 8*,* which is normally what my husband does*,* but like it’s a huge burden on others.’* (P1, F)

‘*I’m…after moving in a nanny. I always have to have a nanny in my house at 7am*,* because if I’m in [redacted*,* hospital] I’m gone at 7:15 [am]. In [redacted*,* hospital]*,* you’re gone at 6:30[am] to be in 6:45[am]. No creche is open*,* you know?’* (P5, F)

The difficulties entailed in starting a family while being geographically distant from a partner were also discussed. Participants detailed basing family planning around rotations, while others discussed delaying starting a family because of either the potential unknowns or not wanting to miss training.

*‘Let’s say you know you’re probably going to be sent somewhere peripheral*,* so again*,* if you’re thinking about pregnancy planning*,* it is in the back of your head like ‘Oh my God*,* like do I try and get pregnant*,* when do I try and get pregnant?’….if you feel like in the following year*,* or the year after*,* that you’re going to get sent to somewhere that’s really difficult for you and your family*,* then that is mainly going to influence the timing of the pregnancy as well.’* (P1, F)

#### Difficulties imbedding into communities

Participants also referenced the difficulties they experienced in imbedding into their communities, whether it be the hospital environment or their broader locality, owing to the regularity with which they were required to rotate. Some participants expressed frustration at the inability to establish themselves in a locality. Trainees detailed being unable to engage in outside interests, with team sport pursuits most commonly cited. In their responses, Trainees detailed losing connection from their teammates and communities due to training.

*‘If you’re involved in a football club or a rugby club*,* or whatever*,* and that’s your life*,* and you’re going every week to training and then suddenly it’s goodbye to everything you know in your community and network and off you go.’* (P8, F)

Trainees also discussed this from a professional standpoint, expressing that trainers were less inclined to invest time in them, owing to their impending next rotation to a different training site. This perception is summarised below by a Trainee, who referenced that, in previous years, Trainees could train in one location, with trainers more likely to invest in what they perceived as the future of their hospital.

*‘I think when you move so often*,* the trainers might have this conversation with a few consultants*,* the trainers don’t get time to invest in you or*,* even if they do*,* they know you’re only there for a year*,* whereas back in the day*,* not saying that back in the day was a better way or anything*,* but back in the day*,* someone might’ve done all their training in one place*,* so they invest in them*,* they see them as their future consultants*,* someone trains them up*,* they have a mentor whereas we don’t really get that because we’re moving so often*.’ (P1, F)

### Managing personal autonomy

#### Living arrangements

There were frequent references in the data to Trainees’ attempts to manage their personal autonomy. This mainly took the form of Trainees detailing their attempts to balance personal and work commitments, with consensus being work took priority. Participants viewed themselves as passive actors in a lottery, unable to exercise individual preference over their training location due to rotating. They detailed being required to make decisions about their living arrangement based on their training schedule and assumption that they would be required to relocate regularly. The cost of maintaining a separate property while rotating was commonly cited by participants in phase 1 and, while this also featured in Phase 2, participants more commonly detailed buying homes in geographically attractive regions, close to linking roads and motorways, with access to potential training locations in mind.

*‘To be honest*,* it’s soul destroying being in this lottery of ‘where am I being sent?’. I honestly would say I worry about it most days of my life. like ‘oh*,* where am I going to go next year and where is my family going to be?’. It’s torture.’* (P1, F)

*‘Myself and my [partner] bought our house in a location that was adjacent to the [motorway] to be able to commute. My job played a part in where we were going to buy. Not where my family live*,* where my support systems are*,* where is reasonable enough that’s close by to the commuter belt.’* (P11, F)

Participants cited a fear of the unknown regarding their training locations and lack of control around same. This is encapsulated by a participant who discussed a potential scenario that could arise as a result of rotating. In their response, they detailed their individual circumstance and the potential domino effect that being relocated could cause, as well as a sense of helplessness they had when required to rotate. detailing the resultant concern this caused them.

*‘My husband is in a job where he could get told in two months’ time that he’s going [overseas*,* redacted] or something for six months and*,* all of a sudden*,* I’m single parenting in a job that’s very inflexible and I could have to move to [a site*,* redacted] in three months’ time*,* away from my parents*,* away from my social supports*,* with my child*,* to try and find childcare for a full time post there. It’s daunting when you think of it….it does play on your mind when you have such little control.’* (P10, F)

#### Commuting

The commute represented an issue for participants, as was identified in the quantitative phase. Participants frequently referenced being required to make long commutes and, while they did discuss the costs associated with commuting, participants most commonly detailed the extent to which their required commute deprived them of personal time. Additionally, the dangers of commuting long distances, particularly after extended shifts were highlighted. Many participants detailed near misses and accidents while driving due to fatigue, expressing dissatisfaction at the requirement to commute rather than being able to exercise their personal preferences over their living situation.

*‘it’s the extra hour*,* or two hours*,* or three hours. Your 39-hour week is now- add 2 hours a day- that’s 49 hours a week because of your commute. Sitting in your car. It’s not enjoyable. It’s costly in terms of finances*,* but it’s costly in terms of the small bit of personal time that we as medical professionals have’.* (P11, F)

*‘It’s also dangerous if you finish a 24-hour shift and you have to drive an hour and a half. I’ve almost fallen asleep on the road.’* (P9, M)

*‘Talking about 24-hour shifts*,* handing over and then getting in the car and driving for an hour home and plenty of us have experienced the near misses*,* some of us have actually had accidents.’* (P5, F)

#### The value placed on training

Despite the difficulties cited by participants regarding rotations, participants constantly referenced the value they personally placed on being present and receiving training. There were frequent references to difficulties associated with being separated from support, with one Trainee highlighting the disconnect between their institution circulating well-being material to Trainees while isolating them socially through rotating. However, the importance of training and attending same was to the forefront. In sum, Trainee’s attitude towards training was that they must attend.

*‘If you’re on a rotation where you feel like you’re going to get loads out of it*,* you’re like*,* ‘Oh*,* that’s a rotation I might not want to miss.’* (P1, F)

*‘Knowing what I know now*,* and where I am now*,* I would still apply for training.’* (P8, F)

## Conclusions

The purpose of this study was to investigate Irish Medical Trainees’ experiences of geographical rotations. A total of 86.2% of participants were required to move from their primary residence on rotation. Of these, 67.6% continued to pay bills associated with their primary residence. The implications of rotations were further explored through focus groups, with participants referencing the schedule and frequency of rotations impacting maintenance of social connections and personal autonomy. Participants commonly expressed that the requirement to relocate removed access to available social support outside of work. This was in line with previous findings where frequent relocating acted as a stressor which depleted internal resources for coping in doctors, which also impeded learning [14]. This observed work-life imbalance, taken with the pre-existing stressors of training in medicine, all impacted Trainee wellbeing [17–19].

As stated, 67.6% of those required to relocate reported having financial outgoings associated with their primary residence while living in secondary accommodation. This was expanded on in the qualitative phase, with outgoings relating to accommodation referenced, in addition to costs associated with childcare and commuting. Respondents frequently referenced the commuting associated with rotating. In a study observing health professionals in a tertiary hospital, longer distances between home and work were significantly associated with a greater risk of accident [19]. Shifts exceeding twenty-four hours were associated with increased risk of motor vehicle crashes, with the authors concluding that driving post extended shift put physicians and other road users at unnecessary risk [19].

The most significant limitation of this study is the sample size. The small sample limits the generalisability of findings. Further, the voluntary nature of participation introduces potential self-selection bias, which may limit the representativeness of the findings. Further limitations include the use of self-report measures and lack of a standardised measure as part of the quantitative phase. To the best of the authors’ knowledge, there is not a measure assessing Medical Trainees’ experiences of geographical rotations, a measure of wellbeing would have provided additional insight into the Trainee experience. The impact of self-report measures was mitigated through the series of focus groups conducted, which provided a holistic view of the Trainee experience. Future research should incorporate a larger sample size, while the addition of a standardised measure would add validity and measurability of the phenomena observed.

In February 2024, the National Taskforce on the Non-Consultant Hospital Doctor (NCHD) Workforce, on behalf of the Department of Health in Ireland, outlined a series of recommendations designed to improve the working and training experience of NCHDs. Among other things, they made a series of recommendations regarding geographical rotations, including that Trainees be provided pre-defined rotations at the outset of the training programme for a minimum of three years [4]. They advised that all postgraduate training programmes establish a geographic structure for training, allowing Trainees to remain in the same geographic area for most of the training programme, further recommending that Trainees only be subject to one rotation requiring living relocation in a 4–5-year scheme [4]. The taskforce acknowledged that some training programmes may not have the appropriate number of Trainees or posts to facilitate a regionalised approach, advising that allocations be reviewed to minimise necessary geographical relocation as much as possible [4]. They advised that an oversight process be established to approve programmes where higher numbers of relocations are required before concluding that the feasibility of providing a national couple matching system for allocation of rotations be explored [4]. This mirrors existing NHS policy where measures to minimise travelling have been successfully put in place^,^ ensuring that geographical rotations are within a Trainee’s own compact training region [20]. As well as this, other options like geographical cohorting limits Trainees to a single location, ensuring improved coordination of care and outcome quality for patients, while inter-deanery transfer allows Trainees to request to train in their preferred location on the basis of a committed relationship, or the breakdown of a committed relationship, as well as physical disability, mental health condition and parental or caring responsibilities [20–22].

This study was the first national investigation into the attitudes and experiences of Medical Trainees undergoing geographical rotations in Ireland and adds significantly to the knowledge base of the Trainee experience in Ireland. These findings capture a shift in training preferences, with flexibility in training arrangements being viewed as more attractive [9, 23]. The inflexibility of current training programmes for Irish Trainees was highlighted, suggesting that making LTFTT more available would have little impact on service provision. Barriers were identified, with inability to access LTFTT posts, career progression and fear of negative perceptions cited, but it was argued that negative perceptions could be mitigated through better dissemination of information about LTFTT and other flexible arrangements.

Though an appetite for LTFTT has been observed in an Irish cohort, posts, as well as financial and institutional support, are limited [24]. Commitments have been made at a governmental level to make fundamental changes to the working conditions of Trainees in Ireland, including creating greater opportunities for flexible arrangements and limiting cross-regional rotations, with growing acknowledgement that the current requirements of Trainees are not being met [10].

## Supplementary Information

Supplementary Material 1.

Supplementary Material 2.
